# Effect of Yttrium on Ce/Ni-Metakaolin Catalysts for CO_2_ Methanation

**DOI:** 10.3390/molecules28207079

**Published:** 2023-10-13

**Authors:** Yuyi Wang, Quan Ye, Xinyu Xu, Abdelghaffar S. Dhmees, Xuemin Cui

**Affiliations:** 1 Guangxi Key Lab of Petrochemical Resource Processing and Process Intensification Technology, School of Chemistry and Chemical Engineering, Guangxi University, Nanning 530004, China; 2114931095@st.gxu.edu.cn (Y.W.); ye-q@st.gxu.edu.cn (Q.Y.); wang-yy@st.gxu.edu.cn (X.X.); 2 Egyptian Petroleum Research Institute, Ahmed El-Zomor St., Nasr City, Cairo 11727, Egypt; abdel_ghffar@yahoo.com

**Keywords:** CO_2_, methanation, metakaolin, catalyst, multiple metals

## Abstract

In recent years, major economies have implemented carbon reduction and carbon neutrality policies. Furthermore, with advancements in science and technology, carbon dioxide (CO_2_) is now considered a valuable raw material for producing carbon-based fuels through hydrogenation. Various concentrations of yttrium (referred to as Y hereafter) were introduced to assess their influence on the catalytic performance of CO_2_ methanation. At a temperature of 300 °C, the catalyst exhibited an impressive CO_2_ conversion rate of 78.4% and maintained remarkable stability throughout a rigorous 100 h stability assessment. The findings suggest that the inclusion of yttrium (Y) promotes the formation of oxygen vacancies and alkaline sites on the catalyst. This, in turn, enhances the reducibility of nickel species, improves the dispersion of nickel particles, and plays a pivotal role in enhancing thermal stability. Furthermore, it offers an innovative design approach for creating highly efficient composite CO_2_ methanation catalysts by controlling particle size and harnessing synergistic catalytic effects at the metal/support interface.

## 1. Introduction

The intensifying Russo–Ukrainian conflict has exacerbated global warming and energy shortages, emphasizing the imperative to tackle the ongoing increase in atmospheric CO_2_ levels caused by persistent fossil fuel consumption [1]. Considering CO_2_’s abundance and cost-effectiveness as a source of carbon and oxygen, there has been a growing impetus to transform CO_2_ into value-added products, thereby mitigating CO_2_ emissions [2]. Academic and industrial research in the field of CO_2_ catalytic hydrogenation is continuously advancing and garnering escalating global interest [3]. CO_2_ hydrogenation presents the potential to produce a diverse array of chemical compounds, such as methane, ethanol, and ethylene [4]. Methane, among these compounds, stands out as a highly promising and widely utilized energy resource [5], rendering the thermodynamically significant CO_2_ methanation reaction of particular significance. In the exhaust gases, CO_2_ captured and renewable H_2_ undergo a catalytic Sabatier reaction during combustion to produce synthetic methane [6].

CO_2_ + 4H_2_ ⇆ CH_4_ + 2H_2_O ΔH = −164 kJ/mol (1)

Over the past few decades, significant time and effort have been devoted worldwide on CO_2_ methanation, with numerous publications reporting on the potential of CO_2_ methanation catalyzed by Ru, Rh, Co, Ni, etc. [7,8,9,10]. Precious metal catalysts manifest remarkable catalytic performance and enhanced specificity for CH_4_. Nonetheless, the high cost of noble metals and their excessively high sintering temperatures have hindered their widespread application, making it challenging to meet the demands of industrial-scale production. Consequently, there has been a growing exploration for low-cost and abundantly available non-noble metal catalysts. The overall efficacy of catalysts is influenced not solely by the active metal, but the characteristics of the support also significantly affect the catalyst’s performance, as they are instrumental in shaping its structure, promoting even distribution, and facilitating the reduction in the metal phase [11,12].

Nickel-based catalysts have garnered significant research interest as a viable substitution for precious metal catalysts in the CO_2_ methanation process, owing to their cost-effectiveness, remarkable activity, and selectivity [13]. At elevated temperatures, nickel-based catalysts, despite their advantages, exhibit susceptibility to carbon deposition and sintering, thus leading to rapid deactivation, and their performance at low temperatures also requires improvement [14]. Consequently, in recent years, the research focus on CO_2_ methanation catalysts has shifted to inhibiting deactivation, exploring promoters, and enhancing low-temperature activity.

Catalyst supports have a vital function in stabilizing the catalyst and preventing the aggregation of active metals. Widely employed supports for CO_2_ methanation catalysts encompass TiO_2_ [15], Al_2_O_3_ [16], ZrO_2_ [17], and others, but these supports are chemically synthesized materials. To introduce a low-cost and environmentally friendly catalyst support, metakaolin, derived from kaolin clay through dehydration at appropriate temperatures (600–900 °C), is an attractive material. Metakaolin, abbreviated as MK, is an activated aluminosilicate (Al_2_O_3_ · 2SiO_2_) and serves as a highly active mineral admixture. Mesoporous solids can be used in the separation of complex mixtures of organic molecules in addition to the proposed use as catalysts in reactions such as CO_2_ methanation [18]. Novia Amalia Sholeha et al. [19] examined the synthesis of nickel catalysts utilizing dealuminated metakaolin zeolite as a supporting material. Metakaolin is not only affordable and readily available but also environmentally friendly, being a clay mineral [20] that facilitates CO_2_ adsorption. Additionally, it exhibits high Al_2_O_3_ and SiO_2_ content, making it a promising candidate as a support for CO_2_ hydrogenation catalysts.

In recent years, CeO_2_ has often been introduced as a second metal in nickel-based catalysts for its notable catalytic enhancing effect in the CO_2_ methanation process. Li et al. [21] uncovered that the incorporation of CeO_2_ suppresses the coalescence of nickel particles and promotes synergy between the active metal and the substrate, thereby enhancing the catalytic effectiveness of Ni catalysts containing nickel. While Ce has attracted attention as a promising promoter when incorporated into nickel-based catalysts for methane conversion, other promoters such as Mg [22,23], Y [24], and Zr [25,26] have also been widely applied for the enhancement of nickel-based catalysts. Researchers have highlighted that Y-doped catalysts based on nickel exhibit dual advantages. They not only enhance the BET surface area, facilitating the creation of weakly basic and moderately basic sites, but also reduce the particle size of nickel, promoting better dispersion. Consequently, Y has demonstrated remarkable efficacy as a catalyst enhancer in the CO_2_ methanation process. It exhibits exceptional promotional properties for catalysts relying on nickel as their active component [27,28]. Furthermore, studies have indicated that Y-modified Co/TiO_2_ enhances the CO_2_ capture capacity and surface basicity of the catalyst’s active species, thus enhancing the catalyst’s activity [29]. Investigations were conducted on Ni-based catalysts doped with Ce-Zr or Ce-Y promoters for various reactions, including methane reforming and methane dry reforming [30,31]. The outcomes reveal that Ni catalysts co-doped with both promoters exhibit enhanced catalytic activity. Nevertheless, previous research primarily focused on Ni-based catalysts modified with only one promoter. Therefore, in pursuit of a highly efficient catalyst, this study incorporates Ce-Y promoters into metakaolin-based nickel catalysts.

To date, there have been no reports on the impact of Ce-Y promotion on the structure, morphology, and CO_2_ conversion rate of nickel-supported metakaolin catalysts in the CO_2_ methanation process.

This knowledge gap necessitates the design of catalysts with superior performance in CO_2_ methanation. This study proposes the utilization of cost-effective, readily available and environmentally friendly metakaolin (MK) as catalyst support. Through a co-impregnation method, Y-Ce/Ni-MK catalysts with a fixed Ni and Ce concentration of 15 wt.% and varying Y loadings (1, 3, and 5 wt.%) were synthesized. Multiple characterization methodologies were utilized to investigate the correlation between catalyst activity and its structure, as well as physicochemical properties. The performance and durability of the Y-Ce/Ni-MK catalysts were explored using a combined gas chromatograph and fixed-bed reactor approach. This investigation explores the impact of Y incorporation on the effectiveness of Ce/Ni-MK catalysts and investigates the synergistic effects arising from the interaction of Y with Ce, and the mechanistic aspects of CO_2_ methanation catalyzed by Y-Ce/Ni-MK catalysts were thoroughly examined.

## 2. Results

### 2.1. Structure of the Catalysts

**Figure 1:**
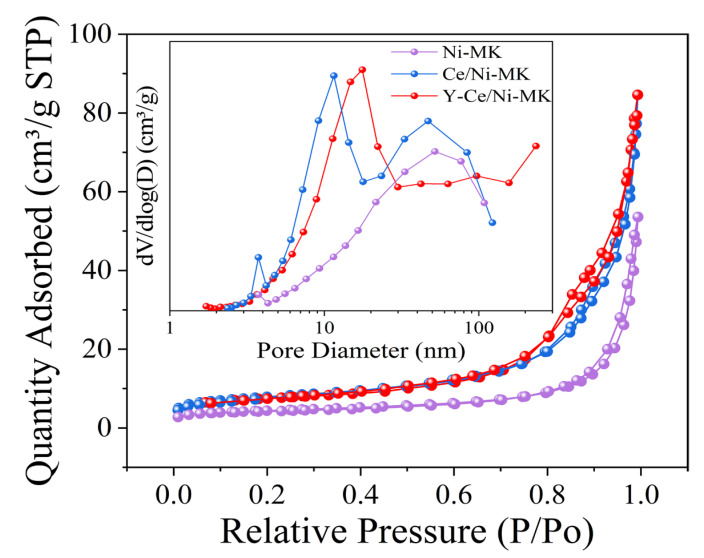
Corresponding BJH pore size distributions and N_2_ adsorption–desorption isotherms for Ni-MK, Ce/Ni-MK, and Y-Ce/Ni-MK catalysts.

As shown in Figure 1, Y-Ce/Ni-MK, Ce/Ni-MK, and Ni-MK all demonstrate type IV adsorption characteristics accompanied by H3-type hysteresis patterns, suggesting the existence of mesoporous architectures in the three catalysts. Data in Table 1 clearly displays that Y-Ce/Ni-MK catalyst outperforms the other two catalysts in terms of specific surface area and pore volume. Due to the existence of mesoporous structures in the catalysts, the cavities within the substrate may experience partial obstruction by Ni following the introduction of the promoter, leading to a marginal decrease in pore dimension for Y-Ce/Ni-MK and Ce/Ni-MK in comparison to Ni-MK.

**Figure 2:**
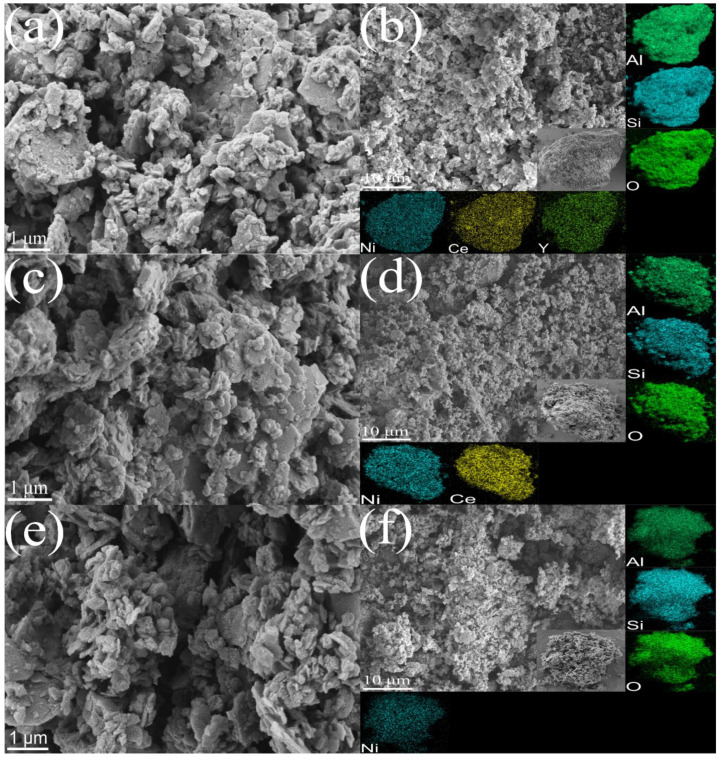
SEM images of the reduced (**a**,**b**) Y-Ce/Ni-MK, (**c**,**d**) Ce/Ni-MK, and (**e**,**f**) Ni-MK catalysts.

The pore structures of the Y-Ce/Ni-MK, Ce/Ni-MK, and Ni-MK samples after reduction are clearly visible in Figure 2. After incorporating active metals and promoters onto MK, the catalyst surfaces manifest a stratified morphology, which is advantageous in augmenting the specific surface area available for reactions. Furthermore, in contrast to nickel-containing catalysts documented in Table 2, the presence of smaller nickel nanoparticles in the catalysts investigated in this study suggests that MK offers a distinct advantage as a support for nickel-based catalysts compared to pure oxides. According to the EDS images, Y_2_O_3_, CeO_2_, and nickel nanoparticles are evenly dispersed on the MK substrate, with the nickel nanoparticles demonstrating excellent dispersion on the Y-Ce/Ni-MK catalyst. Combining Figure 2f with the EDS image of the Ni-MK catalyst reveals that the porosity within the Ni-MK catalyst is blocked, and the exterior is coated with unevenly dispersed metallic nickel.

**Figure 3:**
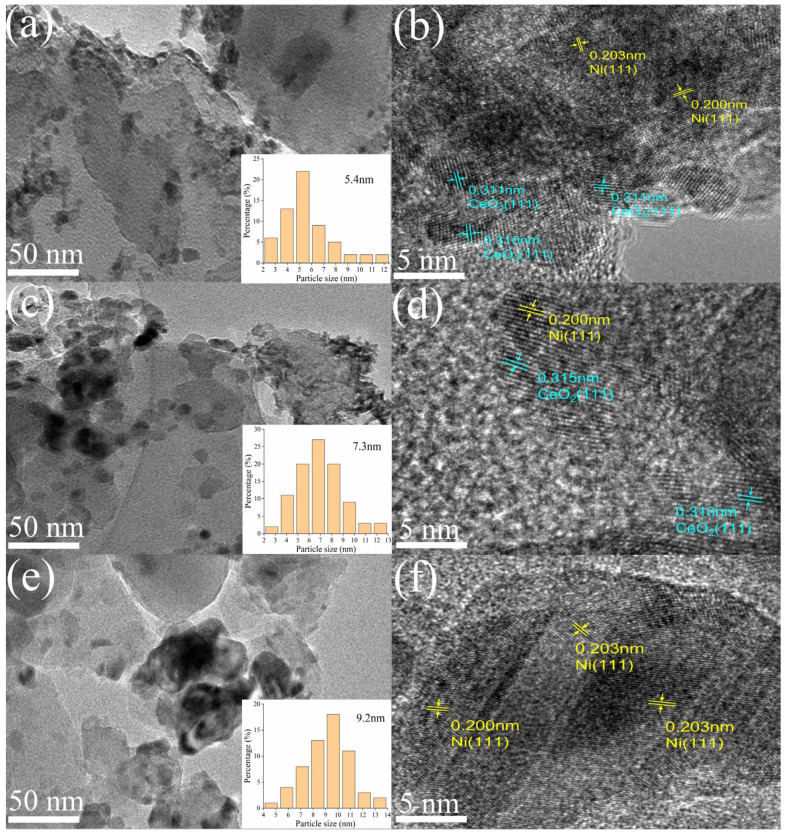
TEM results including mapping and HRTEM images of the reduced Y-Ce/Ni-MK (**a**,**b**), Ce/Ni-MK (**c**,**d**), and Ni-MK (**e**,**f**).

The TEM images (Figure 3) reveal the presence of nanoscale structures in all three catalysts. A comparison between the lattice spacing observed in the HRTEM image of the reduced catalysts and the results obtained from XRD analysis confirms the existence of Ni^0^ and CeO_2_. Furthermore, in the HRTEM image (Figure 3b vs. Figure 3d), the nickel metal is distinctly dispersed and surrounded by CeO_2_, indicating that the incorporation of the Y-Ce promoter leads to a homogeneous distribution of nickel nanoparticles within the CeO_2_ lattice of Y-Ce/Ni-MK [32]. Among the three catalysts, Y-Ce/Ni-MK exhibits the smallest size distribution of reduced Ni nanoparticles, measuring 5.4 nm. Furthermore, the incorporation of CeO_2_ in Ce/Ni-MK results in a smaller size distribution of Ni nanoparticles (7.3 nm) compared to Ni-MK (9.2 nm). This indicates that the addition of CeO_2_ causes a decrease in Ni nanoparticle size and promotes their dispersion. Additionally, the inclusion of Y_2_O_3_ reduces the particle size of CeO_2_ [28], further promoting the dispersion of Ni nanoparticles integrated within the CeO_2_ framework [32] and exerting an inhibitory effect on the movement and aggregation of the Ni nanoparticles.

**Table 2:** Ni loading, Ni nanoparticles, and Ni^0^/(Ni^0^ + Ni^2+^) of various catalysts.

Catalyst	Ni Loading (wt.%)	Ni Nanoparticles (nm)	Ni^0^/(Ni^0^ + Ni^2+^)	Ref.
Ni-MK	15	9.2	5.5	This Work
Ni-CeO_2_	-	17	3.6	[33]
Ni/ZrO_2_-E	15.1	17.46	-	[34]
15Ni/Al_2_O_3_	15	17.2	-	[35]
Ni/Al_2_O_3_-P	15	12.5	-	[36]
Ni/MgO	10	12	-	[37]
Ni-P-SGS	15	13.2	-	[38]

### 2.2. Metal Distribution and Chemical Surface Composition of the Studied Catalysts

**Figure 4:**
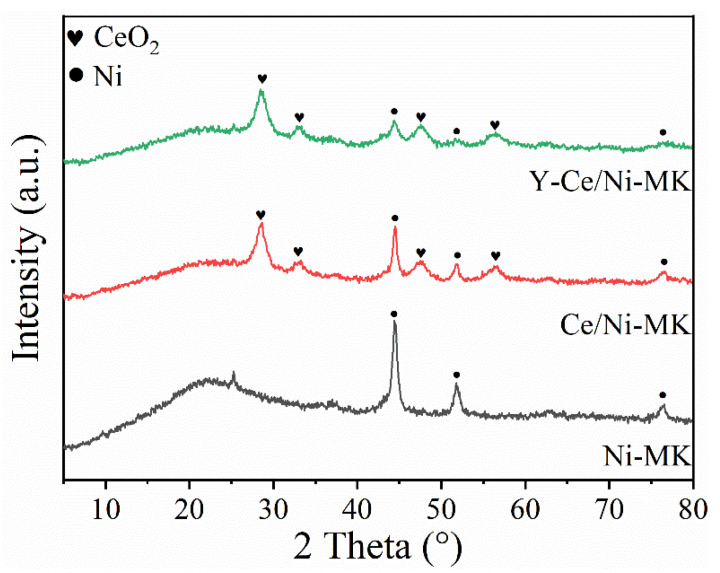
XRD patterns of the reduced Y-Ce/Ni-MK, Ce/Ni-MK, and Ni-MK catalysts.

Based on the characterization results from wide-angle XRD, the reduced samples exhibit broad and amorphous scattering peaks, which are characteristics of highly dispersed materials. The diffraction peaks observed at 44.5°, 51.8°, and 76.4° correspond to the crystal lattice of metallic nickel (JCPDS#87-0712). Significantly, when the incorporation of metallic nickel reaches 15%, the diffraction peaks corresponding to Ni exhibit pronounced and enhanced intensity, suggesting an elevated level of crystallinity. However, upon incorporating Y metal, the strength of the Ni diffraction signals gradually diminishes, implying that the doping of Y improves the dispersibility of Ni and reduces the Ni grain size [38]. Moreover, in Figure 4, it is challenging to identify the diffraction peaks corresponding to the Y species, indicating their high dispersion on the MK matrix [39]. Meanwhile, for the Y-modified Ni/Ce-based catalyst, the diffraction peaks of CeO_2_ in the 2θ range of 27° to 30° do not show significant changes upon Y doping. This suggests that the doping of Y positively affects the structural robustness of the catalyst [40]. In summary, these findings illustrate that the incorporation of Y contributes favorably to decreasing the Ni particle size and improving the dispersion of Ni.

**Figure 5:**
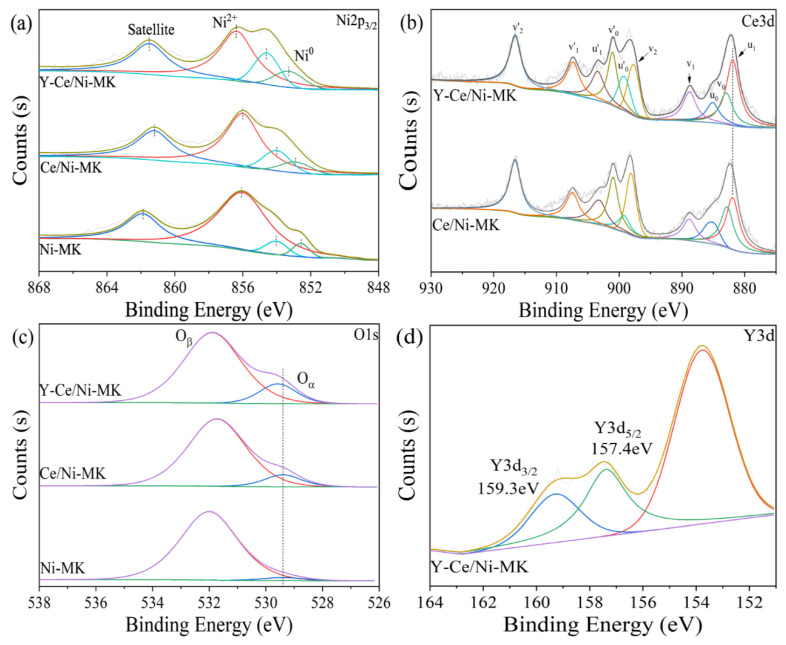
XPS spectra of the reduced Y-Ce/Ni-MK, Ce/Ni-MK, and Ni-MK catalysts; (**a**) Ni2p3/2, (**b**) O1s, (**c**) Ce3d, (**d**) Y3d.

To assess the surface composition of the catalyst and the metal oxidation states, XPS examination was conducted on the catalyst after reduction. Illustrated in Figure 5a, the XPS graph of Ni2p_3/2_ obtained from the Y-Ce/Ni-MK catalyst exhibits Gaussian peaks positioned at 853.3, 854.5, and 856.2 eV, signifying the presence of Ni^0^, NiO, and Ni(OH)_2_, in that order. The 861.5 eV peak corresponds to the shake-up satellite feature [41,42]. The occurrence of Ni^2+^ can be linked to the incomplete oxidation of the catalyst while being exposed to air throughout the transfer process after reduction in a tubular furnace. Both Ce/Ni-MK and Ni-MK exhibit similar four peaks, but the binding energy of Y-Ce/Ni-MK is higher compared to the other two. Additionally, based on quantitative atomic energy ratio analysis (as shown in Table 3), the addition of Y_2_O_3_ increases the ratio of Ni^0^/(Ni^0+^Ni^2+^) from 5.5% to 10.2% for the Y-Ce/Ni-MK catalyst. The incorporation of Y results in an increased extent of Ni^0^ reduction, enhancing the abundance of active sites and subsequently improving the catalytic activity.

Through the process of deconvolution, it becomes possible to identify two distinct oxygen species present within the energy range of 528–533 eV, as indicated by the O1s peak. The spectral region ranging from 528 to 530 eV exhibits a peak indicative of oxygen originating from the crystal lattice, denoted as Oα, while the peak observed within the energy range of 530–533 eV can be ascribed to surface oxygen that is loosely bound and chemisorbed. The examination of Table 3 reveals that the Y-Ce/Ni-MK catalyst showcases the utmost quantity of lattice oxygen. As shown in Figure 5d, the Y^3+^ species is represented by two peaks in the Y3d orbitals at 157.4 eV (Y3d_5/2_) and 159.3 eV (Y3d_3/2_), consistent with other literature [40]. This indicates a weak interaction between Y and Ce, resulting in no significant peak shift for Y^3+^.

To further characterize the valence states of Ce3d, a deconvolution analysis was performed, resulting in ten peaks. Peaks u_1_, u_0_, u’_1_, and u’_0_ were assigned to Ce^3+^, while peaks v_0_, v_1_, v_2_, v’_0_, v’_1_, and v’_2_ were associated with Ce^4+^. Based on Table 3, it is evident that the Y-Ce/Ni-MK catalyst displays a higher percentage of Ce^3+^ (42.0%) compared to the Ce/Ni-MK catalyst (32.6%), indicating an increase of approximately 28.8%. This indicates that as a result of the likeness in ionic sizes between Ce and Y and their proximity, Ce^4+^ is substituted by Y^3+^, thereby boosting the level of Ce^3+^ and generating additional oxygen vacancies. During the reaction progression, this process positively affects the increase in Ce^3+^ concentration and the formation of additional oxygen vacancies facilitated by Y^3+^ [43]. As a result, the capacity of the carrier to reduce nickel entities is strengthened owing to the augmented quantity of oxygen vacancies, thereby resulting in enhanced catalytic activation performance.

### 2.3. Catalyst Reducibility and Adsorption Property

**Figure 6:**
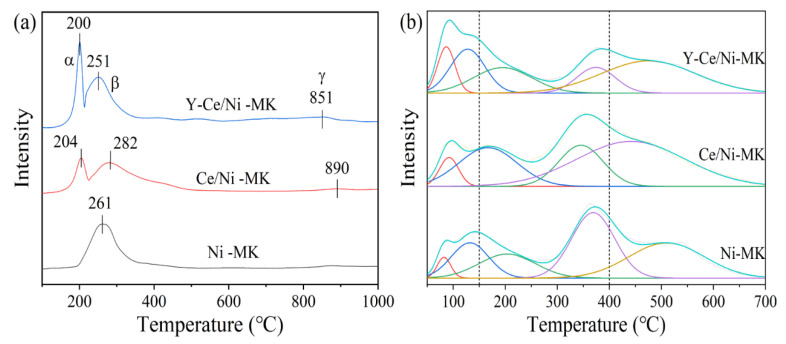
(**a**) H_2_-TPR and (**b**) CO_2_-TPD results of the reduced Y-Ce/Ni-MK, Ce/Ni-MK, and Ni-MK catalysts.

H_2_-TPR experiments were conducted on the three catalysts across a temperature span from 50 to 1000 °C to investigate the interaction between Y_2_O_3_ and NiO, CeO_2_, as well as the catalysts’ reducing abilities. The H_2_-TPR curves are shown in Figure 6, while Table 4 presents the peak temperatures and values for hydrogen consumption. In the graph, the Y-Ce/Ni-MK catalyst showed three reduction peaks, designated as α, β, and γ. The relatively weak γ peak appearing at (T > 800 °C) indicates the reduction of surface-bound lattice oxygen in CeO_2_ [44]. The limited interaction between NiO and the MK carrier, as indicated by its larger particle size, can be attributed to the presence of the β peak, which corresponds to the reduction of NiO [33]. The infiltration of Y^3+^ or Ni^2+^ within the CeO_2_ matrix, leading to the formation of Y-Ce-O or Ni-Ce-O homogeneous phases, surplus oxygen vacancies are generated. Consequently, the appearance of the α peak can be ascribed to the reduction of oxide entities attached to these vacancies [40]. With the addition of Y, the intensities of α and β peaks increase and shift towards lower temperatures. Research findings indicate a correlation between the reduction temperature and the dimensions of NiO particles, wherein smaller particle sizes correspond to reduced temperatures for the reduction process [45]. Within the context of this experiment, the introduction of Y element demonstrates a decrease in the temperature at which the main peaks undergo reduction, an augmentation in the catalyst’s capability to store hydrogen, a decrease in the dimensions of NiO particles, and a reinforcement in the interplay between multimetallic species and the carrier. It is noteworthy to highlight that the hydrogen utilization associated with the α peak (as shown in Table 4) exhibits an upward trend upon the introduction of Y. Meanwhile, the hydrogen consumption corresponding to the β peak also shows a corresponding increase as a result of the heightened existence of oxygen vacancies. Nevertheless, the utilization of hydrogen for the β + γ peaks experiences an increase in the Y-Ce/Ni-MK catalyst. This suggests that Y-Ce/Ni-MK catalyst produces more oxygen vacancies in CeO_2_, exhibiting stronger oxygen chemisorption capacity.

CO_2_-TPD analyses were performed on the three catalysts to examine their surface basicity and adsorption capacity in relation to the CO_2_ methanation process. By analyzing the CO_2_ desorption curves, distinct temperature ranges were identified for the desorption of CO_2_: the weak region (<150 °C), the medium region (150–400 °C), and the strong region (>400 °C) [46,47]. Figure 6 illustrates the desorption behavior of CO_2_ through the CO_2_-TPD curves of the three catalysts. A comprehensive evaluation, involving the measurement and assessment, is provided in Table 5, presenting data on the occurrence and distribution of alkaline sites determined through the integrated desorption peaks technique [48]. According to the data in Table 5, The inclusion of Y_2_O_3_ leads to an increase in the overall quantity of alkaline sites present in the catalysts, and the Y-Ce/Ni-MK catalyst exhibits the highest total basicity among the three catalysts. With the addition of Y_2_O_3_, the following observations can be made from Table 5: (I) there is an escalation in the count of potent alkaline sites; (II) compared to the Ce/Ni-MK catalyst, there is a small reduction in the quantity of moderate alkaline sites; and (III) there is a notable rise in the quantity of weak alkaline sites. The critical role played by the coordination of moderate and weak alkaline sites in the adsorption of CO_2_ is essential for enhancing its strong adsorption at low temperatures and promoting the catalyst’s activity at those temperatures [49,50,51]. The movement of CO_2_ desorption peaks to reduced temperatures in the Y-Ce/Ni-MK catalyst demonstrates the exceptional synergistic effects between Y_2_O_3_, CeO_2_, active metallic constituents, and the MK carrier, facilitating the CO_2_ activation at low temperatures [13]. To summarize, the addition of Y_2_O_3_ elevates the overall basicity of the catalyst, specifically augmenting the potency of moderate and weak alkaline sites, thereby amplifying the catalyst’s capacity to capture and transform CO_2_.

### 2.4. In Situ DRIFTS Analysis

**Figure 7:**
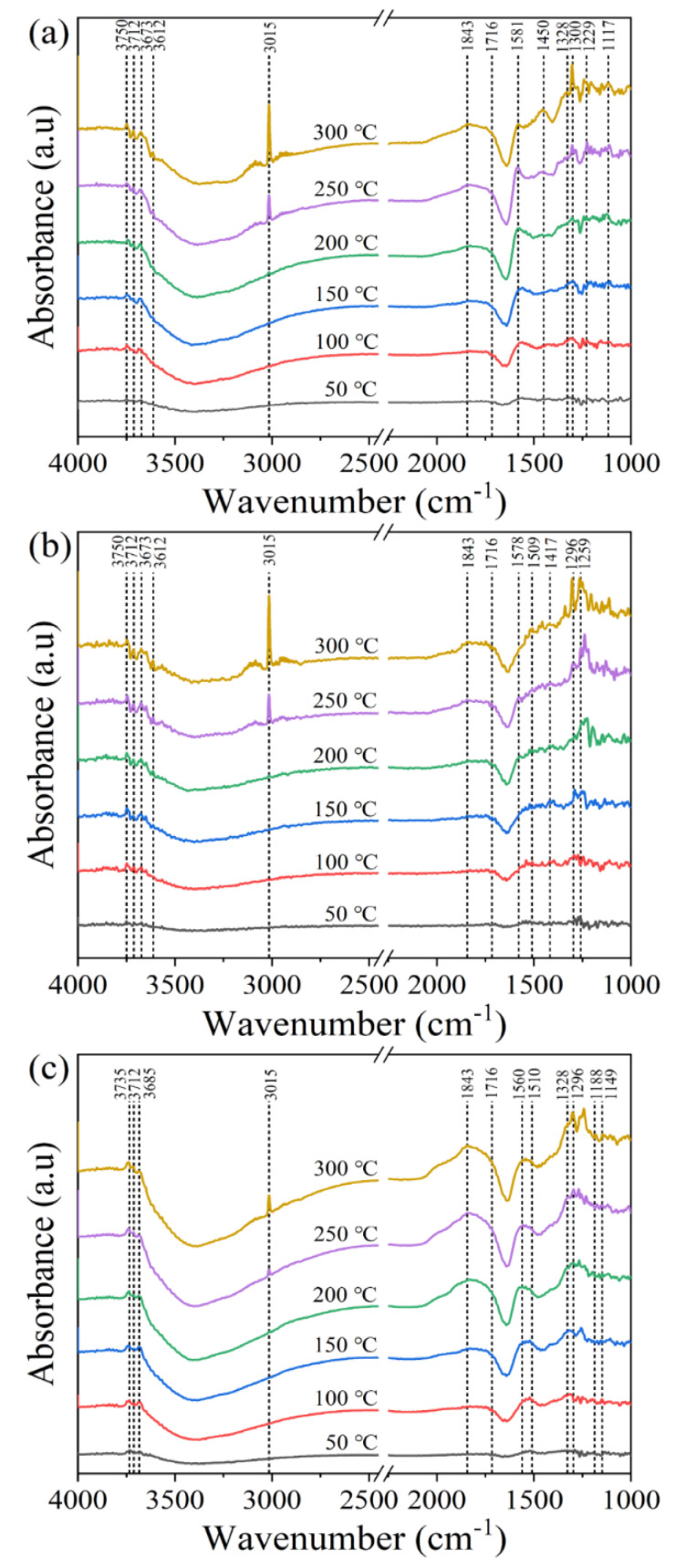
In situ DRIFTS investigation of CO_2_ methanation over the all catalysts: (**a**) Y-Ce/Ni-MK, (**b**) Ce/Ni-MK, and (**c**) Ni-MK catalysts.

To elucidate the working principle of the catalysts’ performance, in situ DRIFTS analysis was conducted on the Y-Ce/Ni-MK, Ce/Ni-MK, and Ni-MK samples. The objective of this analysis was to examine the species involved in the methanation of CO_2_ and investigate the pathways of the reaction. As shown in Figure 7, a characteristic CH_4_ peak (3015 cm^−1^) was observed for all three catalysts between 200 and 300 °C, with the peak intensity increasing with the addition of the promoter and the temperature elevation. Among them, the Y-Ce/Ni-MK catalyst exhibited the highest peak intensity, indicating a rapid CH_4_ formation. Various OH peaks were observed in the infrared spectrum between 3612 and 3800 cm^−1^, which declined as the temperature increased. Evident from Figure 7b,c, monodentate carbonates (1417 cm^−1^, 1328 cm^−1^) and bidentate carbonates (1578 cm^−1^, 1510 cm^−1^) were observed in the spectrum, with monodentate carbonates gradually transforming into monodentate formates (1296 cm^−1^) with increasing temperature [46,52]. The absence of substantial migration and transformation of bidentate formates within the Ce/Ni-MK and Ni-MK catalysts is apparent, indicating their resistance to conversion. As shown in Figure 7a, monodentate carbonates (1328 cm^−1^) and bidentate carbonates (1581 cm^−1^) of the Y-Ce/Ni-MK catalyst were observed in the spectrum, and with increasing temperature, they gradually transformed into monodentate formates (1300 cm^−1^) and bidentate formates (1450 cm^−1^). The reason behind this phenomenon can be traced to the elongation and flexion oscillations taking place on the initial O-C-O groups of formats [53,54]. Formates are key intermediates in the methanation process. Although the Ce/Ni-MK and Ni-MK catalysts produce monodentate formates, the conversion rate and intensity of monodentate and bidentate formates on the Y-Ce/Ni-MK catalyst exhibit a notable superiority compared to the remaining two catalysts. In conclusion, the Y-Ce/Ni-MK catalyst enhances the activity of carbonate conversion to formate and improves the ability to rapidly form CH_4_, thereby exhibiting higher catalytic activity.

**Figure 8:**
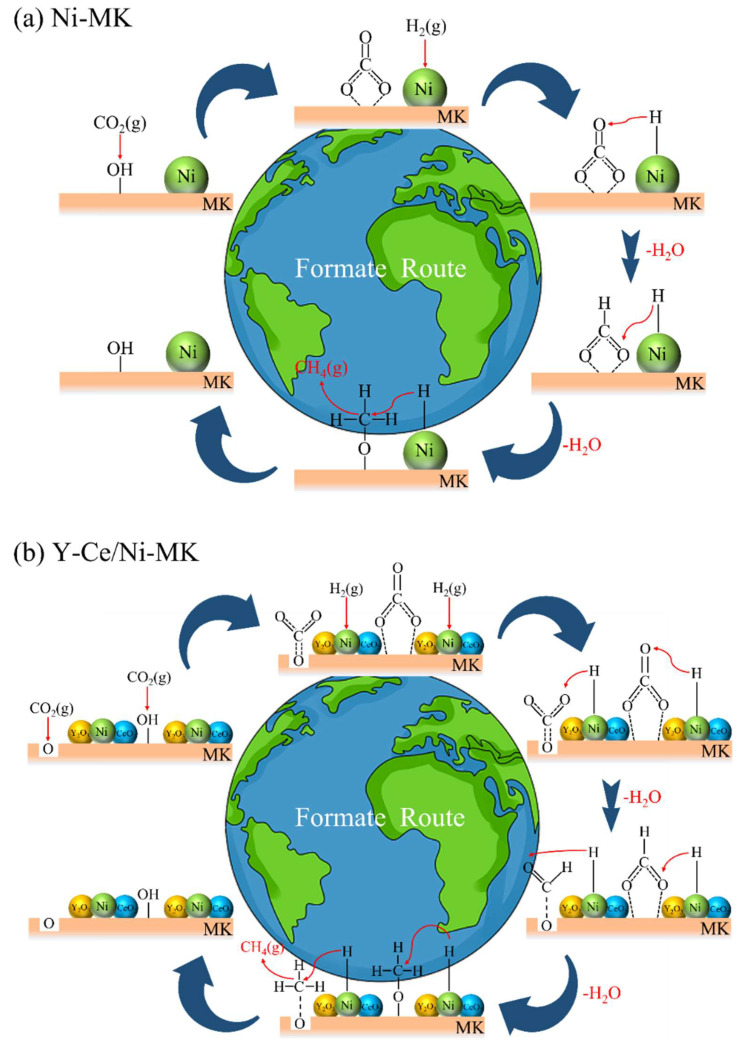
The main proposed pathway for CO_2_ methanation over (**a**) Ni-MK and (**b**) Y-Ce/Ni-MK catalysts.

Based on current investigations, it has been revealed that the methanation process of CO_2_ encompasses two distinct reaction pathways. One pathway entails the direct interaction between formates and H_2_, resulting in the formation of methane, while the other pathway involves the dissociation of formates into CO, followed by hydrogenation into methane [55]. However, the in situ DRIFTS measurements of the three catalysts did not exhibit any distinctive CO infrared absorption features. Consequently, our conjecture is that the reaction mechanisms of the three catalysts encompass the direct conversion of formates through hydrogenation, leading to the production of methane, as depicted in Figure 8. At the catalyst surface, CO_2_ initially undergoes adsorption and reacts with hydroxyl (OH) groups, resulting in the formation of carbonates. The Ni nanoparticles attract H_2_ and dissociate it into atomic hydrogen. Subsequently, the carbonates react with the atomic hydrogen to form formates and water. Finally, the formates react with hydrogen to produce methane.

### 2.5. Catalytic Performance of All Catalysts and Stability Test

**Figure 9:**
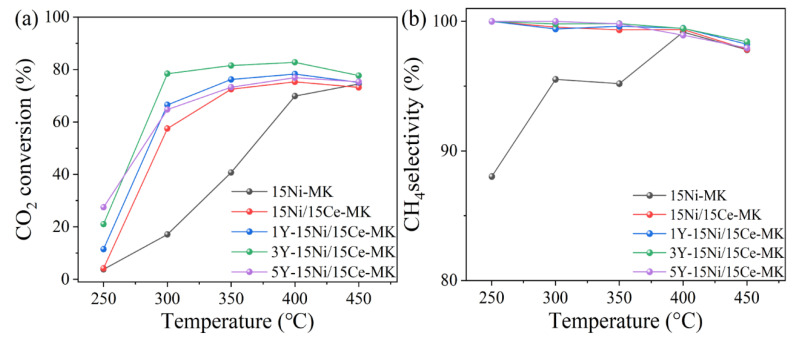
The Y-Ce/Ni-MK, Ce/Ni-MK, and Ni-MK catalysts synthesized in this study exhibited (**a**) CO_2_ conversion and (**b**) CH_4_ selectivity.

At a pressure of 1.0 bar and with an airflow rate of 12,000 mL·g^−1^·h^−1^, the performance of each catalyst was evaluated over a temperature range spanning from 250 °C to 450 °C. As depicted in Table 6, Y-Ce/Ni-MK exhibits superior catalytic activity at low temperatures compared to other catalysts reported in the literature. According to the data presented in Figure 9, compared to Ce/Ni-MK and Ni-MK, the Y-Ce/Ni-MK catalyst exhibited superior activation performance with the addition of Y under conditions of reduced temperature at 300 °C and atmospheric pressure. At 300 °C, the CO_2_ conversion rate reached 78.4%, while the corresponding CH_4_ selectivity was 99.8%. At equivalent theoretical Ni loading, the Y-modified Y-Ce/Ni-MK catalyst demonstrated enhanced catalytic efficiency, surpassing both Ce/Ni-MK and Ni-MK in terms of activity at lower operating temperatures. The achievement allowed for achieving high CO_2_ conversion rates without the need for high temperatures (above 400 °C), thereby reducing energy consumption.

The key to achieving low-temperature high-performance with the Y-Ce/Ni-MK catalyst lies in the addition of Y_2_O_3_, which enhances the concentration of oxygen vacancies, increases the strength of weak alkaline sites on the catalyst, and promotes the reactivity of CO_2_. This addition contributes positively to enhancing the CO_2_ conversion efficiency. Moreover, the inclusion of Y_2_O_3_ restricts the diffusion of metal surface free energy, improves the dispersion of Ni, and contributes to the augmentation of catalytic performance by offering an increased number of active metal sites.

**Figure 10:**
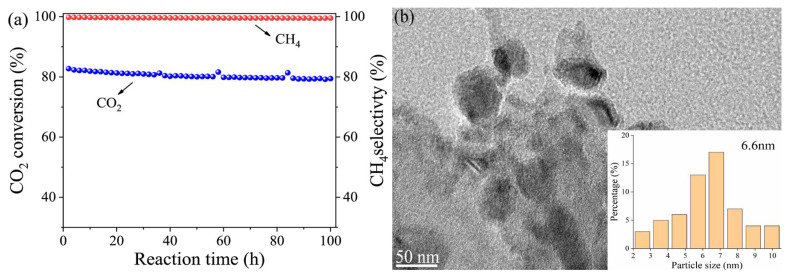
(**a**) The stability of the Y-Ce/Ni-MK catalysts over the long term; (**b**) HRTEM results of the reduced Y-Ce/Ni-MK.

The stability over extended periods is essential for the successful commercialization of any catalyst. Therefore, stability tests were performed on the catalyst samples of Y-Ce/Ni-MK that exhibited optimal performance. The catalyst underwent a stability test for a duration of 100 h, employing the following reaction conditions: the pressure set at 0.1 MP, the temperature maintained at 400 °C, and a flow rate of 12,000 mL·h^−1^·g^−1^. The catalyst’s CH_4_ selectivity and CO_2_ conversion rate are depicted within Figure 10a. Throughout the 100 h test, the catalyst exhibited a decrease in its CO_2_ conversion efficiency of approximately 3.9%, while the CH_4_ selectivity exhibited no changes. Figure 10b displays the TEM image depicting the Y-Ce/Ni-MK catalyst following the 100 h stability test. Only a minor aggregation of nickel particles was observed, resulting in a rise in the mean diameter of Ni particles from 5.4 nm to 6.6 nm. In summary, the MK-based catalyst exhibits excellent stability during long-term catalysis, and the addition of Y-Ce initiators synergistically enhances the low-temperature activation performance of the MK support, while simultaneously improving the catalyst’s stability over an extended duration.

## 3. Materials and Methods

### 3.1. Catalyst Preparation

Metakaolin, derived from kaolin clay, is a dehydrated aluminosilicate formed through constant calcination at temperatures ranging from 500 to 800 °C. Its detailed composition is listed in Table 7. The high-activity metakaolin utilized in this study was procured from Inner Mongolia Chaopai Kaolin Co., Ltd. (Togrogul Town, China), and exhibits a white appearance.

The x wt% Y-Ni/Ce-MK catalysts (where x = 1, 3, 5) were synthesized using the impregnation technique, loading equimolar amounts of metal Ni and Ce onto the metakaolin (MK) support, along with varying amounts of metal Y. For convenience, these catalysts are abbreviated as xY-Ce/Ni-MK. Hexahydrated nickel nitrate (Ni(NO_3_)_2_·6H_2_O) and hexahydrated cerium nitrate (Ce(NO_3_)_3_·6H_2_O) (>99.0%, Macklin Chemicals, Shanghai, China) were dissolved in deionized water at room temperature. Different mass percentages of Y(NO_3_)_3_·6H_2_O, a high-purity yttrium nitrate hexahydrate from Macklin Chemicals (>99.5%), were introduced into the aforementioned solution. The mixture was then sonicated for 15 min to attain a homogeneous dispersion. Then, the metal solution was dropped onto the MK support (5.00 g) in a beaker. The mass percentage of Y relative to Ce/Ni-MK was altered at 1, 3, and 5 wt.%, while the loading of Ni and Ce remained fixed at 15 wt.% across all catalysts. After the synthesis, the obtained samples were kept at ambient conditions for 24 h and subsequently subjected to drying at 100 °C for a duration of 24 h. The specimens were desiccated, crushed, and sifted through a 100–120 mesh sieve to acquire powders with a Y content of 3 wt.%, along with 15 wt.% Ce and 15 wt.% Ni, denoted as Y-Ce/Ni-MK. As a control experiment, Ce/Ni-MK and Ni-MK samples were synthesized using the identical procedure, with metal loadings of 5 wt.% Ce + 15 wt.% Ni and 15 wt.% Ni, respectively.

### 3.2. Catalytic Activity

The catalysts were assessed for their CO_2_ methanation performance in a fixed-bed reactor operating under continuous flow conditions. The operating pressure was consistently maintained at 0.1 MPa, while an embedded K-type thermocouple, located inside a stainless-steel tube, was employed as a temperature monitoring system to oversee the temperature within the catalyst bed. Placing 200 milligrams of catalyst into a fixed-bed reactor tube measuring 450 mm in length and 8 mm in width, securing the catalyst at both ends using quartz wool. Before conducting the activity tests, the samples underwent a 2 h reduction process. The samples were subjected to a reduction process at a temperature of 500 °C and a pressure of 0.1 MPa. This process involved exposing the samples to a gas mixture containing H_2_ and N_2_, with a molar ratio of 1:9. (space velocity, WHSV = 12,000 mL g^−1^ h^−1^). Once the catalysts were cooled to ambient temperature, a methane reaction was conducted using a feed gas with a CO_2_/H_2_ ratio of 1:4. The gas product composition at the exit was evaluated by means of a gas analyzer furnished with a thermal conductivity detector (GC9800), and the amounts of each component gas were measured. To investigate the catalyst activity, the temperature range of 200 °C to 450 °C was employed during the experimental procedure. After a 30 min dwelling time at each 50 °C interval, stable measurements were obtained by introducing the samples. The gas compositions at the outlet were analyzed using an internal reference technique.

The catalyst’s efficiency was evaluated based on its effectiveness in converting CO_2_ and selecting CH_4_. The ratio of consumed CO_2_ to the initial CO_2_ feedstock during the reaction was used to calculate the CO_2_ conversion efficiency. The calculation for the CH_4_ selectivity and CO_2_ conversion rate was as outlined below:

(2) $$X_{CO_{2}}=\frac{n_{CO_{2}}^{in}-n_{CO_{2}}^{out}}{n_{CO_{2}}^{in}}\;\times 100\%$$

(3) $$S_{CH_{4}}=\frac{n_{CH_{4}}^{in}}{n_{CH_{4}}^{out}+n_{CH_{4}}^{out}}\times 100\%$$

where $n_{CO_{2}}^{in}$ represents the CO_2_ flow rate within the feed-stream, and $n_{CO_{2}}^{out}$ and $n_{CH_{4}}^{out}\;$ indicate the flow velocities of CO_2_ and CH_4_ within the reformed gas at the outlet.

### 3.3. Catalyst Characterization

X-ray diffraction (XRD) analysis was conducted on the catalysts utilizing a Rigaku MiniFlex 600 instrument (Tokyo, Japan) for diffraction measurements, with emitted radiation source of Cu-Kα (a wavelength of 0.154 nm). It operated with a voltage of 40 kV and a current of 15 mA. The span of measurement, encompassing from 20° to 80°, was selected, employing a step size of 5° per minute.

To determine the actual concentrations of Ni, Ce, and Y within the catalyst, inductively coupled plasma optical emission spectroscopy (ICPOES) was performed using an Agilent 720ES(OES) instrument (Santa Clara, CA, USA).

The catalyst samples underwent an 8 h pretreatment in a Micromeritics APSP 2460 (Norcross, GA, USA) degassing station at 300 °C under vacuum. Subsequently, the measurement of nitrogen adsorption–desorption isotherms was conducted using the same instrument at 77 K (liquid nitrogen temperature). To determine the overall specific surface area of the catalyst, the obtained isotherms were analyzed using the Brunauer–Emmett–Teller (BET) technique. The Barrett–Joyner–Halenda (BJH) technique was employed for the calculation of the pore size and pore volume of the samples.

The Micromeritics AutoChem II 2920 device (Norcross, GA, USA) was utilized to perform the hydrogen thermal ramp reduction (H_2_-TPR) experiment. Initially, the catalyst was subjected to drying and pretreatment at 300 °C with a catalyst mass of 30 mg. Subsequently, the catalyst underwent a helium flow (50 mL/min) for a duration of 1 h, followed by cooling it down to 50 °C. After that, in the presence of a flow of 10% H_2_/Ar (50 mL/min), the specimen was subjected to a temperature ramping process at a rate of 10 °C/min, reaching up to 1000 °C during desorption.

The CO_2_ temperature-programmed desorption (CO_2_-TPD) tests were performed using the Micromeritics AutoChem II 2920 instrument. Initially, a 50 mg catalyst sample was carefully weighed and introduced into the reaction tube. Subsequently, the catalyst underwent a drying pretreatment by gradually increasing the temperature from ambient conditions to 300 °C. After a duration of 1 h, the system was subsequently cooled down to 50 °C while a helium flow (30–50 mL/min) was directed over the catalyst. Subsequently, a blend comprising carbon dioxide and helium gas (30–50 mL/min) was introduced for 1 h until saturation, after which the helium flow (30–50 mL/min) was switched to remove weakly adsorbed CO_2_ on the catalyst surface for 1 h. In the final step, with a controlled increment of 10 °C per minute, the temperature was raised to 700 °C in the presence of a helium environment. During this process, by utilizing a thermal conductivity detector (TCD), the desorption profiles were recorded, ensuring precise temperature monitoring and collection of desorption data.

X-ray photoelectron spectroscopy (XPS; Thermo Scientific K-Alpha; Al Κα X-ray source) was employed to calibrate the oxidation states of each element, using C1s at 284.8 eV as the reference.

A field-emission scanning electron microscope (FE-SEM) model SU8220 from Hitachi (Tokyo, Japan), along with an energy-dispersive X-ray spectroscopy (EDX) system provided by Bruker, was employed for scanning analysis and elemental composition analysis of the reduced catalyst.

Using a FEI 80-300 (West Springfield, MA, USA) Titan (S) transmission electron microscope (HRTEM) operating at 300 kV, the catalyst samples were inspected to observe their morphology, and the size of metal particles was determined through the utilization of the Nano Measurer (1.2) software.

During the reaction, the formation of intermediate species was investigated, in situ diffuse reflectance Fourier-transform infrared spectroscopy (DRIFTS) was utilized. Using a Nicolet IS 50 spectrometers equipped with a cryogenically cooled MCT detector, in situ DRIFTS measurements were conducted. Before conducting the measurements, the sample was subjected to a reduction process at a temperature of 500 °C for a period of 2 h, during which it was subjected to a stream of hydrogen (30 mL min^−1^). The sample, which had undergone reduction, weighing 5 mg, was positioned on the sample holder, precisely at the midpoint of the sample pool. Afterward, a blend of carbon dioxide and hydrogen was introduced, and the thermal condition was elevated at a speed of 5 °C per minute until reaching 300 °C, with an infrared spectrum captured at every 50 °C increment.

## 4. Conclusions

In this study, a novel Y-Ce/Ni-MK catalyst was synthesized by employing metakaolin as a support via the co-impregnation method. The inclusion of yttrium (Y) had beneficial effects, promoting the creation of oxygen vacancies and alkaline sites. This played a pivotal role in improving the reducibility of nickel species, enhancing nickel particle dispersion, and increasing thermal stability. The catalyst demonstrated outstanding catalytic performance at approximately 300 °C and retained its high activity throughout a 100 h stability test. These results underscore the substantial potential of environmentally friendly, cost-effective metakaolin-supported nickel-based catalysts. These catalysts, prepared using a straightforward method, are promising for CO_2_ methanation at lower temperatures. The investigation unveiled synergistic interactions between the Y-Ce promoter, the Ni catalyst, and the metakaolin substrate, resulting in increased activity in the CO_2_ methanation reaction. This study provides an economical and straightforward method for producing stable catalysts for CO_2_ methanation, characterized by high activity. It explores the potential of synergistic catalysis involving multiple metals and metakaolin support, offering a greener, more environmentally friendly, and cost-efficient alternative for industrial CO_2_ methanation applications.

## Figures and Tables

**Table 1:** Physicochemical attributes of Ni-MK, Ce/Ni-MK, and Y-Ce/Ni-MK.

Sample	Surface Area (m^2^·g^−1^)	Pore Volume (cm^3^·g^−1^)	Pore Diameter (nm)	Ni Loading (wt.%) ^a^
Ni-MK	15.8	0.0828	20.8	13.64
Ce/Ni-MK	26.8	0.1227	18.2	12.96
Y-Ce/Ni-MK	28.3	0.1309	18.5	12.92

^a^ Determined by ICP-OES.

**Table 3:** XPS deconvolution results of Ni, O, and Ce.

Sample	Binding Energy(eV)				Atomic Ratio (%)		
	Ni2p_3/2_	Ce3d_5/2_	Os1	Y3d_5/2_	Ni^0^/(Ni^0^ + Ni^2+^)	Ce^3+^/(Ce^3+^+Ce^4+^)	O_α_/(O_α_ + O_β_)
Ni-MK	856.0	–	529.4	–	5.5	–	3.9
Ce/Ni-MK	856.0	881.9	529.4	–	7.7	32.6	10.5
Y-Ce/Ni-MK	856.2	881.9	529.5	157.4	10.2	42.0	15.3

**Table 4:** H_2_-TPR results for catalysts.

Sample	Temperature (℃)			H_2_ Consumption (μmol/g) ^a^			Total H_2_ Uptake (μmol/g) ^a^
	α	β	γ	α	β	γ	
Ni-MK	-	261	-	-	1654.0	-	1654.0
Ce/Ni-MK	216	259	-	486.3	1698.5	-	2184.8
Y-Ce/Ni-MK	200	251	851	687.3	1820.5	-	2507.3

^a^ Determined by H_2_-TPR.

**Table 5:** CO_2_-TPD results for catalysts.

Sample	Weak Basic Sites (μmol/g) ^a^	Medium Basic Sites (μmol/g) ^a^	Strong Basic Sites (μmol/g) ^a^	Total Basicity (μmol/g) ^a^
Ni-MK	60.5	168.3	135.8	364.6
Ce/Ni-MK	60.7	204.1	147.6	412.4
Y-Ce/Ni-MK	165.0	190.5	170.3	525.8

^a^ Determined by CO_2_-TPD.

**Table 6:** CO_2_ methanation activities of various catalysts.

Catalyst	H_2_:CO_2_	Space Velocity (mL·g^−1^⋅h^−1^)	CO_2_ Conversion (%)	CH_4_ Selectivity (%)	Reaction Temperature (℃)	Ref.
Y-Ce/Ni-MK	4:1	12,000	78.4	99.8	300	This Work
25Ni-5Ce-Al_2_O_3_	3.5:1	9000	72	98	300	[56]
Ni-CP-V2.0	4:1	12,000	68	96	300	[57]
15Ni10Ce10Y/SBA-15	4:1	12,000	20	97	300	[39]
20Ce/15Ni/CsUSY(38)	4:1	-	66	98	300	[58]
HEO-4-900/H_2_	4:1	9000	62	98	300	[59]

**Table 7:** Chemical composition of metakaolin.

Chemical Composition (Oxide)	SiO_2_	Al_2_O_3_	Fe_2_O_3_	K_2_O	Na_2_O	MgO
Metakaolin (wt.%)	48.75	42.34	0.48	0.089	0.39	0.13

## Data Availability

Not applicable.
